# Inflammation-inducing Factors of *Mycoplasma pneumoniae*

**DOI:** 10.3389/fmicb.2016.00414

**Published:** 2016-03-31

**Authors:** Takashi Shimizu

**Affiliations:** Laboratory of Veterinary Public Health, Joint Faculty of Veterinary Medicine, Yamaguchi UniversityYamaguchi, Japan

**Keywords:** mycoplasma, lipoprotein, cytadherence, inflammation, pneumonia

## Abstract

*Mycoplasma pneumoniae*, which causes mycoplasmal pneumonia in human, mainly causes pneumonia in children, although it occasionally causes disease in infants and geriatrics. Some pathogenic factors produced by *M. pneumoniae*, such as hydrogen peroxide and Community-Acquired Respiratory Distress Syndrome (CARDS) toxin have been well studied. However, these factors alone cannot explain this predilection. The low incidence rate of mycoplasmal pneumonia in infants and geriatrics implies that the strong inflammatory responses induced by *M. pneumoniae* coordinate with the pathogenic factors to induce pneumonia. However, *M. pneumoniae* lacks a cell wall and does not possess an inflammation-inducing endotoxin, such as lipopolysaccharide (LPS). In *M. pneumoniae*, lipoproteins were identified as an inflammation-inducing factor. Lipoproteins induce inflammatory responses through Toll-like receptors (TLR) 2. Because *Mycoplasma* species lack a cell wall and lipoproteins anchored in the membrane are exposed, lipoproteins and TLR2 have been thought to be important for the pathogenesis of *M. pneumoniae*. However, recent reports suggest that *M. pneumoniae* also induces inflammatory responses also in a TLR2-independent manner. TLR4 and autophagy are involved in this TLR2-independent inflammation. In addition, the CARDS toxin or *M. pneumoniae* cytadherence induces inflammatory responses through an intracellular receptor protein complex called the inflammasome. In this review, the inflammation-inducing factors of *M. pneumoniae* are summarized.

## Introduction

*Mycoplasma pneumoniae* causes primary atypical pneumonia, tracheobronchitis, pharyngitis, and asthma in humans (Gil et al., 1993; Kraft et al., 1998; Waites and Talkington, 2004). The age distribution of patients with pneumonia caused by *M. pneumoniae* is characteristic. The incidence is highest among school-aged children and young adults and lower in infants and geriatrics (Denny et al., 1971; Foy et al., 1979). Some pathogenic factors of *M. pneumoniae*, such as hydrogen peroxide, Community-Acquired Respiratory Distress Syndrome (CARDS) toxin, and nuclease, have been reported to be associated with the development of pneumonia (Somerson et al., 1965; Cohen and Somerson, 1967; Kannan and Baseman, 2006; Hames et al., 2009; Somarajan et al., 2010). However, these pathogenic factors are insufficient to explain the age distribution of patients with pneumonia caused by *M. pneumoniae*. Generally, the immune system in infants and geriatrics is immature compared with that in young adults. The symptoms of pneumonia caused by *M. pneumoniae* are correlated with the induction of pro-inflammatory cytokines (Tryon and Baseman, 1992; Salvatore et al., 2007). These findings suggest that the excessive immune responses induced by *M. pneumoniae* play an important role in the development of pneumonia. In this review, the molecular mechanisms of inflammation induced by *M. pneumoniae* are summarized (**Table 1**).

**Table 1:** Summary of the inflammation-inducing factors of *Mycoplasma pneumoniae.*

Gene ID	Original function	Function in inflammation
MPN602	F_0_F_1_ ATP synthase subunit b	Diacylated lipoprotein
MPN052	*Hypothetical	4*Triacylated lipoprotein
MPN162		
MPN415		
MPN611		
MPN141	Cytadherence, P1 adhesin	Pro-inflammatory cytokine induction
MPN142	Cytadherence, P40, P90	5*Activation of inflammasome
MPN453	Cytadherence, P30	
MPN447	Cytadherence, HMW1	
MPN372	ADP-ribosylating toxin, CARDS toxin
MPN333	ABC transporter	Autophagy/TLR4 dependent
MPN597	F_0_F_1_ ATP synthase subunit ε	inflammation

## Lipoproteins and Toll-Like Receptors (TLRs)

### Lipoproteins of *Mycoplasma* Species

It has been reported that some *Mycoplasma* species induce pro-inflammatory cytokines and stimulate various immune cells (Atkin et al., 1986; Kirchner et al., 1986; Teh et al., 1988). Because *Mycoplasma* species are devoid of a cell wall and lack immune cell stimulator such as lipopolysaccharide (LPS) or peptidoglycan (Mizel et al., 1978; Staber et al., 1978), the factors responsible for the induction of inflammatory responses have been unclear for a long time. The first report on the inflammation-inducing factor of *Mycoplasma* species was published by Quentmeier et al. (1990). They reported that a high-molecular-weight (HMW) protein of *M. fermentans* known as MDHM possesses interleukin (IL)-6-inducing activity in macrophages. Because the activity of MDHM was resistant to proteinase K, the active component of MDHM was thought to be a low-molecular-weight compound. In 1996, Muhlradt et al. identified the active component of MDHM as *S*-(2,3-dihydroxypropyl) cysteine (Muhlradt et al., 1996). This component was similar to the N-terminal structure of an *Escherichia coli*-derived lipoprotein identified in 1969 by Braun et al. Braun (1975). Muhlradt et al. (1997) also purified the inflammation-inducing factor from *M. fermentans* culture and demonstrated that the active component is the diacylated lipopeptide, *S*-(2,3-bisacyloxypropyl)-CGNNDESNISFKEK. They named it macrophage-activating lipopeptide-2 (MALP-2). After these reports, inflammation-inducing lipoproteins were purified and identified in various *Mycoplasma* species (Jan et al., 1996a; Muhlradt et al., 1997, 1998; Shibata et al., 2000), including *M. pneumoniae* (Shimizu et al., 2005).

### Structure of Lipoprotein and TLR

**FIGURE 1:**
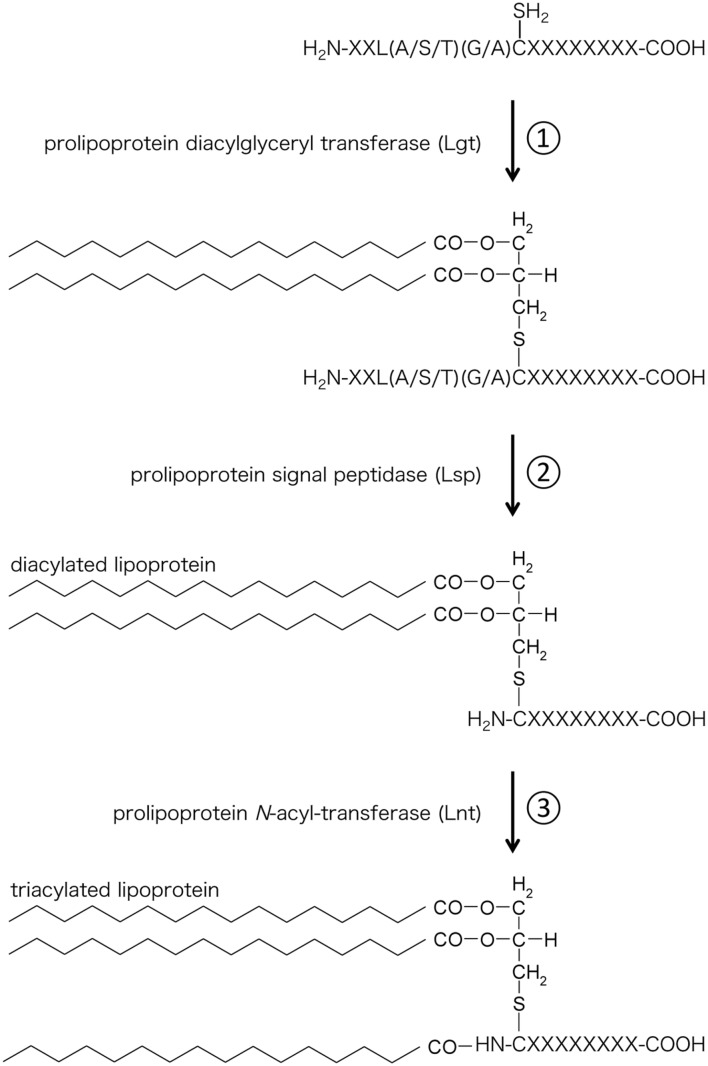
**Biosynthesis of bacterial lipoproteins. (1)** Lgt transfers a diacylglyceryl moiety from a phospholipid to the sulfhydryl group of the cysteine located after the lipobox sequence. **(2)** Lsp cleaves the signal peptide at the N-terminus of the cysteine. **(3)** Lnt transfers an acyl chain derived from phospholipid to the amino group of the cysteine.

Lipoproteins were discovered in 1969 by Braun et al. (Braun, 1975). Lipoproteins are hydrophilic membrane proteins characterized by a conserved N-terminal lipid-modified cysteine residue. Lipoproteins contain *S*-glyceryl cysteine modified with three fatty acids (*N*-acyl-*S*-diacylglyceryl cysteine) at their N-terminal. This triacylated structure is also called Braun’s lipoprotein. Braun’s Lipoproteins are synthesized by the following three steps (**Figure 1**): (1) Transfer of the diacylglyceryl moiety from a membrane phospholipid to a cysteine residue of a protein through the recognition of the lipobox (L-[A/S/T]-[G/A]-C) by prolipoprotein diacylglyceryl transferase (Lgt); (2) Digestion of the signal sequence at the amino-terminal side of the cysteine by prolipoprotein signal peptidase (Lsp); and (3) Linkage of an acyl chain to the amino group of the amino-terminal cysteine (*N*-acylation) by prolipoprotein *N*-acyl-transferase (Lnt). Because genes orthologous to Lnt gene are not found in some bacterial species (Firmicutes and and Tenericutes), including *Mycoplasma* species, lipoproteins from these bacterial species have been assumed to be of the diacylated form (Nakayama et al., 2012).

Toll-like receptors are a type of pattern-recognition receptors that play critical roles in early innate recognition and host inflammatory responses against invading microbes (Kopp and Medzhitov, 1999; Akira and Takeda, 2004). Among the 11 reported TLR family members, TLR2 plays a central role in the recognition of lipoproteins. TLR2 recognizes the *S*-diacylglyceryl cysteine portions of both diacylated and triacylated lipoproteins (Botos et al., 2011). TLR1 coordinate with TLR2 to recognize triacylated lipoproteins through the recognition of the third acyl chain amide-linked to the cysteine using a hydrophobic pocket within TLR1 (Jin et al., 2007). However, TLR2 alone is not able to recognize diacylated lipoproteins. TLR6 assists in the recognition of diacylated lipoproteins by associating with the amino acid portion of diacylated lipoproteins (Kang et al., 2009). Although there are some exceptions (Buwitt-Beckmann et al., 2005; Kurokawa et al., 2009), diacylated and triacylated lipoproteins are generally recognaized by TLR2/6 and TLR2/1 heterodimers, respectively.

The presence of triacylated lipoproteins in *Mycoplasma* species has been controversial. The lipoproteins from *M. fermentans*, *M. hyorhinis*, *M. salivarium*, and *M. gallisepticum* have been shown to be diacylated lipoproteins and not *N*-acylated (Jan et al., 1996a; Muhlradt et al., 1997, 1998; Shibata et al., 2000). The Lnt gene, which encodes the enzyme responsible for N-acylation has not been found in any mycoplasma genomes (Fraser et al., 1995; Himmelreich et al., 1996; Sasaki et al., 2002). However, a study on the ratio of *N*-amide and *O*-ester bonds in *M. gallisepticum* and *M. mycoides* suggested the presence of triacylated lipoproteins (Jan et al., 1996b). Furthermore, the resistance to Edoman degradation of proteins from *M. mycoides* also indicated the presence of *N*-acylation (Chambaud et al., 1999). These data imply the presence of triacylated lipoproteins in *Mycoplasma* species.

### Lipoproteins of *M. pneumoniae*

Lipoproteins of *M. pneumonie* are summarized in **Table 2**. In *M. pneumoniae*, Shimizu et al. (2005) reported that the subunit b of the F_0_F_1_ ATP synthase (MPN602) is a diacylated lipoprotein that induces inflammatory responses through TLR2. Into et al. (2007) also reported that *M. pneumoniae* has 48 lipoproteins and that the common N-terminal structure of these lipoproteins induces inflammatory responses. Interestingly, Some of these lipoproteins (MPN162, MPN611) were recognized by TLR1 and TLR2, suggesting that *M. pneumoniae* contains triacylted lipoproteins (Shimizu et al., 2007). Kurokawa et al. (2012) analyzed the detailed structure of *M. pneumoniae* lipoproteins using lipoprotein lipase-based mass spectrometry analysis, and demonstrated that some of *M. pneumoniae* lipoproteins (MPN052, MPN415) are triacylated. In this study, triacylated lipoproteins were also found in *M. genitalium*. These findings led to the conclusion that *Mycoplasma* species possess triacylated lipoproteins and indicated that a new enzyme with Lnt activity exists in *Mycoplasma* species. Although the modification of other 43 lipoproteins of *M. penoumoniae* is still unclear, the lipoproteins of *M. pneumoniae* seem mixture of diacylated and triacylated lipoproteins. Induction of inflammatory responses through both TLR2/6 and TLR2/1 by diacylated and triacylated lipoproteins may affect the strong inflammation in *M. pneumoniae* infection.

**Table 2:** Summary of lipoproteins of *M. pneumoniae.*

Gene ID	Gene symbol	Original function	Usage of TLR	Number of acyl chain
MPN011		Hypothetical		
MPN052		Hypothetical		3^a^
MPN054		Hypothetical		
MPN058		Hypothetical		
MPN083		Hypothetical		
MPN097		Pseudo		
MPN133		Hypothetical		
MPN152		Hypothetical		
MPN162		Hypothetical	1, 2	3^b^
MPN199		Hypothetical		
MPN200		Hypothetical		
MPN271		Hypothetical		
MPN281		Pseudo		
MPN284		Hypothetical		
MPN288		Hypothetical		
MPN363		Hypothetical		
MPN369		Hypothetical		
MPN408		Hypothetical		
MPN411		Hypothetical		
MPN415		High affinity transport system protein P37		3^a^
MPN436		Hypothetical		
MPN439		Pseudo		
MPN442		Hypothetical		
MPN456		Hypothetical		
MPN459		Hypothetical		
MPN467		Hypothetical		
MPN489		Hypothetical		
MPN506		Hypothetical		
MPN523		Hypothetical		
MPN582		Hypothetical		
MPN585		Hypothetical		
MPN587		Hypothetical		
MPN588		Hypothetical		
MPN590		Hypothetical		
MPN592		Hypothetical		
MPN602	atpF	F_0_F_1_ ATP synthase subunit b	2, 6	2^b^
MPN611		Phosphate ABC transporter substrate-binding protein	1, 2	3^b^
MPN639		Hypothetical		
MPN640		Hypothetical		
MPN641		Hypothetical		
MPN642		Hypothetical		
MPN643		Hypothetical		
MPN644		Hypothetical		
MPN645		Hypothetical		
MPN646		Hypothetical		
MPN647		Hypothetical		
MPN650		Hypothetical		
MPN654		Hypothetical		

*^a^Determined by lipase-based mass spectrometry analysis.*

*^b^Estimated from TLR usage.*

## TLR2-Independent Inflammation

### *M. pneumoniae* and Autophagy

Because *Mycoplasma* species lack cell walls, they do not contain immunostimulants such as LPS, peptidoglycan, or lipoteichoic acid. Therefore, lipoproteins seem to be key factors in *M. pneumoniae*-induced inflammatory responses and to facilitate the development of pneumonia in humans. However, the existence of lipoproteins in non-pathogenic *Mycoplasma* species suggests the presence of an alternative mechanism by which *M. pneumoniae* induce inflammatory responses.

Autophagy is a cellular response that involves the sequestration of regions within the cytosol with double membrane compartments. Autophagy has been shown to play important roles in the cellular response to starvation, cell death, removal of damaged organelles, and neurodegenerative diseases (Levine, 2005). It has recently been recognized that autophagy is involved in both innate and adaptive immunity against various microorganisms (Schmid and Munz, 2007; Deretic et al., 2013; Ma et al., 2013).

Recently, Shimizu et al. demonstrated that *M. pneumoniae* induces strong inflammatory responses, even in macrophages derived from TLR2 knockout (KO) mice (Shimizu et al., 2014). *M. pneumoniae* internalized into macrophages through phagocytosis were co-localized with the autophagosome, and autophagy inhibitors decreased the induction of pro-inflammatory cytokines, suggesting the autophagy-mediated induction of inflammatory responses. Because this TLR2-independent induction was inhibited in macrophages derived from TLR2/4 double KO mouse, TLR4 is also involved. In this study, they also reported that the ABC-transporter (MPN333), and F_0_F_1_ ATP synthase subunit ε (MPN597) of *M. pneumoniae* are essential for the activation of the autophagy/TLR4-mediated pathway.

### *M. pneumoniae* and the Inflammasome

Inflammasomes are intracellular receptors (Martinon et al., 2009), that respond to various signals, including intracellular bacterial toxins, pathogen-associated molecular patterns (PAMPs) (Martinon et al., 2004; Miao et al., 2007), damage-associated molecular patterns (DAMPs) (Kanneganti et al., 2006; Mariathasan et al., 2006; Sutterwala et al., 2006), and reactive oxygen species (Dostert et al., 2008; Allen et al., 2009). Activated inflammasomes cleave the precursors of pro-inflammatory cytokines, such as IL-1β and IL-18 through caspase-1 or caspase-11, and release them (Boyden and Dietrich, 2006).

Shimizu et al. (2011) reported that *M. pneumoniae* induces eﬄux of ATP from host cells. The eﬄux of ATP activated inflammasomes via the P2X7 receptor, which is followed by the secretion of IL-1β. A recent report by Sugiyama et al. (2015) also demonstrated that *M. pnumoniae* induces IL-1β through the NLRP3 inflammasome in a dendritic cell line.

Interestingly, Bose et al. (2014) showed that CARDS toxin (MPN372) regulates NLRP3 inflammasome activity. CARDs toxin is a vacuolating cytotoxin produced by some *Mycoplasma* species, including *M. pneumoniae*. Its C-terminal region is responsible for its vacuolating activity (Kannan and Baseman, 2006; Kannan et al., 2014). Its N-terminal region shares sequence similarity with pertussis toxin and is essential for its ADP-ribosylating activity. In this study, they demonstrated that CARDS toxin activates inflammasomes through the ADP-ribosylation of NLRP3 and enhances the secretion of IL-1β.

Taken together, these findings suggest that inflammasomes play an important role in the inflammation induced by *M. pneumoniae*.

### Cytadherence of *M. pneumoniae* and Inflammation

Cytadherence property is one of the unique characteristics of *M. pneumoniae.* Cytadherence in the respiratory tract, the initial event in *M. pneumoniae* infection, is mediated by P1 (MPN141) adhesin and other accessory proteins, such as P30 and HMW proteins (Krause and Balish, 2001; Balish and Krause, 2002; Miyata, 2008a,b). The relationship between cytoadherence and the induction of inflammatory responses was first reported in Yang et al. (2002). They demonstrated that protease treatment or anti-P1 antibody treatment decreases the induction of pro-inflammatory cytokines, including IL-1β. Hoek et al. (2005) reported that culturing *M. pneumoniae* in polypropylene bottles reduces the expression of P1 adhesin. Under these conditions, the induction of IL-4 from mast cells was significantly decreased. As described above, Shimizu et al. reported that *M. pneumoniae* induces the eﬄux of ATP from host cells, followed by the activation of inflammasomes and secretion of IL-1β. In this study, they also reported that cytadherence-deficient mutants lacking P90 and P40 (MPN142, 130 kDa precursor) or HMW1 and P30 (MPN447 and MPN453, respectively) fail to induce IL-1β through ATP eﬄux. Cytadherence was also associated with autophagy/TLR4-mediated induction of inflammatory responses. Mutation in ABC-transporter (MPN333), and F_0_F_1_ ATP synthase subunit ε (MPN597) failed to induce inflammatory responses, and these mutants showed a deficiency in cytadherence (Shimizu et al., 2014). Taken together, these findings indicate that cytadherence of *M. pneumoniae* is strongly associated with the induction of inflammatory responses.

## Conclusion

**FIGURE 2:**
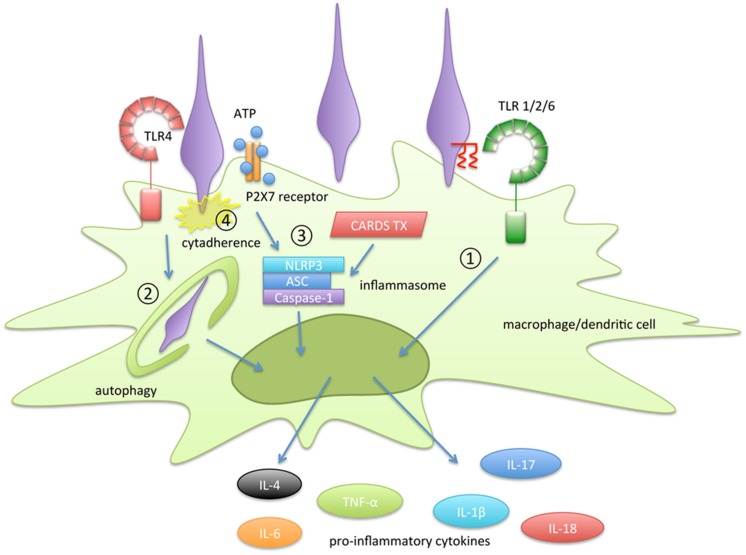
**Summary of the inflammation-inducing pathways in *Mycoplasma pneumoniae* infection.** The following four pathways are involved in the induction of inflammatory responses: **(1)** recognition of lipoprotein by TLR2, **(2)** autophagy-mediated signaling, **(3)** activation of inflammasomes, and **(4)** cytadherence property.

In this review, the molecular mechanisms of inflammatory responses induced by *M. pneumoniae* were reviewed (**Figure 2**). The following four pathways are important for the induction of inflammatory responses in *M. pneumoniae* infection: 1) recognition of lipoprotein by TLR2, 2) autophagy-mediated signaling; 3) activation of inflammasomes, and 4) cytadherence property. Lipoproteins, which were the first immunostimulants discovered in *Mycoplasma* species, have been well studied. However, the structures of the lipoproteins in *Mycoplasma* species are identical to those of lipoproteins from other bacteria, including normal microflora. Therefore, lipoproteins alone are insufficient to explain the inflammatory responses induced by *M. pneumoniae*. *M. pneumoniae* also has the ability to induce inflammatory responses through a TLR2-independent pathway. Autophagy and TLR4 are involved in this induction. Some pro-inflammatory cytokines, such as IL-1β and IL-18, are matured and released through inflammasome activation. Inflammasome activation is necessary to release these cytokines during *M. pneumoniae* infection. It is noteworthy that CARDS toxin enhances inflammasome activation. The distribution of CARDS toxin in *Mycoplasma* species is limited to a small number of *Mycoplasma* species. In addition, cytadherent property of *M. pneumoniae* is strongly associated with the autophagy/TLR4- and inflammasome- mediated induction of inflammatory responses. Although some *Mycoplasma* species, such as *M. genitalium* and *M. gallisepticum*, have partially similar adhesin, cytadherence mediated by P1 adhesin is unique in *M. pneumoniae*. These characteristics may contribute to the greater ability of *M. pneumoniae* to induce inflammatory responses than non-pathogenic *Mycoplasma* species.

In addition to inflammation-inducing factors, *M. pneumoniae* shows cytotoxicity through CARDS toxin, nuclease, and hydrogen peroxide produced during glycerol metabolism. The symptoms of mycoplasmal pneumonia, such as fever and severe cough, are thought to appear as a result of a combination of inflammation and cytotoxicity induced by *M. pneumoniae*. Mycoplasmal pneumonia is still an important issue in the field of pediatric medicine. Although measures to prevent mycoplasmal pneumonia are desired worldwide, preventive measures, including vaccines, have not been developed. Therefore, the inflammation-inducing factors of *M. pneumoniae* described here may be suitable targets for the development of new preventive measures.

## Author Contributions

The author confirms being the sole contributor of this work and approved it for publication.

## Conflict of Interest Statement

The author declares that the research was conducted in the absence of any commercial or financial relationships that could be construed as a potential conflict of interest.
